# Rapid Evaluation of Gastric Content With Ultrasound: An Educational Tool

**DOI:** 10.7759/cureus.49031

**Published:** 2023-11-18

**Authors:** Huynh Nguyen, Matthew R Paluska, Ricardo Falcon, Timothy R Petersen, Codruta Soneru

**Affiliations:** 1 Department of Medicine, The Keck School of Medicine of the University of Southern California, Los Angeles, USA; 2 Department of Anesthesiology, Rocky Vista University College of Osteopathic Medicine, Englewood, USA; 3 Department of Anesthesiology and Critical Care, University of New Mexico School of Medicine, Albuquerque, USA; 4 Office of Graduate Medical Education, University of New Mexico School of Medicine, Albuquerque, USA; 5 Department of Obstetrics & Gynecology, University of New Mexico School of Medicine, Albuquerque, USA

**Keywords:** anesthesiology, aspiration risk, gastric content, gastric ultrasound, preoperative assessment, ultrasound

## Abstract

Pulmonary aspiration is a severe complication in patients receiving anesthesia for surgical procedures. The risk and severity of aspiration are significantly higher in the presence of substantial gastric contents. Bedside ultrasound imaging of the gastric antrum is emerging as a rapid and valuable method to evaluate gastric contents before surgery. Rapid gastric ultrasound using a three-category grading system promotes timely decision-making to help in emergent or urgent surgeries by identifying patients with potentially high gastric volumes or solid food contents. In emergent cases with limited time, a single ultrasound view of the gastric antrum is still likely to yield helpful information. In this report, we argue that bedside ultrasound offers a more reliable assessment of gastric contents than assumptions based on time-based fasting guidelines.

## Introduction

Food or liquids present in the stomach before the induction of anesthesia substantially elevate the risk of pulmonary complications such as aspiration during the perioperative period [1]. Pulmonary aspiration is a serious complication, characterized by the presence of secretions or particulate matter within the tracheobronchial tree. Aspiration often arises from the passive regurgitation of gastric contents or active vomiting during anesthesia induction, leading to the inadvertent inhalation of these materials into the airways and lungs [2]. In normal physiologic contexts, anatomy such as the gastroesophageal junction and upper esophageal sphincter, as well as upper airway reflexes (e.g., coughing, expiration, and laryngospasm), prevent pulmonary aspiration [3]. General anesthesia increases the risk of this complication due to both the suppression of these protective reflexes with the loss of consciousness, and the loss of tone of the upper esophageal sphincter during the induction process [4].

Aspiration is considered a 'never event' in clinical practice; thus, its avoidance has highly shaped the standard of care as outlined in the American Society of Anesthesiologists (ASA) guidelines. It is a significant concern for patients undergoing anesthesia for elective or emergent procedures, as it is responsible for numerous adverse anesthesia events [5], and occult gastric content is a significant risk factor for this occurrence [6]. Aspiration can result in severe morbidity and possible mortality via likely caustic pneumonia, sometimes necessitating prolonged ventilation [7]. In surgical patients, aspiration is associated with a four-fold increased risk of intensive care admission, a nine-day increase in length of stay, and a 7.6-fold increased risk of in-hospital mortality [1]. Preoperative fasting, as recommended by *nil per os* (NPO) guidelines from the ASA, is central to mitigating aspiration risk and prioritizing patient safety. The ASA Practice Guidelines for Preoperative Fasting recommend that healthy patients be NPO for a minimum of two hours for clear liquids; four hours for human breastmilk; six hours for non-human breastmilk, infant formula, or light meals; and eight hours for fried foods, fatty foods, and meat [8,9].

However, these guidelines are often generalized, failing to discriminate between ambulatory and inpatient settings and thereby resulting in unnecessarily prolonged fasting [10]. Patient factors such as delayed gastric emptying or situational factors such as emergency surgery may also make common time-based fasting guidelines less helpful [7]. Delayed gastric emptying can accompany pain, previous gastrointestinal surgery, opioid therapy, viral gastroenteritis infection, and uncommon conditions like neurological disorders and mesenteric ischemia [7,11]. Recent research has called traditional fasting protocols into question, indicating that a more liberal approach to clear fluid intake, such as allowing fluids up to one hour before surgery, does not increase the incidence of adverse events [10,12-16].

A reevaluation of fasting guidelines is likely warranted, particularly given the adverse effects of prolonged fasting, such as hemodynamic instability, patient discomfort, and extended hospital stays [10]. Advancements in gastric ultrasound offer a promising solution. The ASA Practice Guidelines for Preoperative Fasting emphasizes fasting periods for different food types but does not address high-risk patients. Gastric ultrasound could fill this gap, offering a personalized approach to assessing aspiration risk. This technique requires an understanding of the stomach's anatomy and neighboring organs but enables a real-time, non-invasive assessment of gastric contents.

There are four commonly recognized regions of the stomach: the cardia, fundus, body, and pylorus [17]. Located just after the esophagus is the cardia. The fundus, situated above the cardiac notch, represents the uppermost region of the stomach. The body, being the largest region, serves as the primary area for mixing food and the action of digestive enzymes. The funnel-like pylorus is at the stomach's distal end, which includes the antrum and the pyloric sphincter. The antrum, marking the transition between the stomach's body and the pylorus, plays a crucial role in storing food before it passes through the pyloric sphincter into the duodenum [17]. Notably, due to its orientation and location in the pyloric region, the antrum is a critical anatomical feature in gastric ultrasound examinations.

Point-of-care gastric ultrasound is a rapidly evolving imaging technique known for its reliability, accuracy, ease of use, and repeatability [18]. It provides detailed qualitative and quantitative insights into the gastric contents. This modality can be instrumental at the bedside in acute care settings for aspiration risk evaluation when there is clinical uncertainty.

## Technical report

This technical report synthesizes insights gained from the authors' experience with over 400 gastric ultrasound examinations conducted since 2012 [Abstract: Reviere A, Falcon R, Davis DD, Petersen T, Soneru C. When is NPO status not good enough? An observational study of pediatric long bone fracture patients' gastric contents. Presented at: Society for Pediatric Anesthesia/American Academy of Pediatrics' Pediatric Anesthesiology 2022 Meetings; 2022].

Bedside gastric ultrasound

Almost all diagnostic ultrasound machines are suitable for performing a gastric ultrasound [1]. Bedside gastric ultrasound immediately before surgery can be a valuable tool to assess gastric contents [6,7,19], and the gastric antrum appears to be the most valuable single location [6,7,20]. This portion of the stomach is readily identified on ultrasound and offers a more significant likelihood of fully imaging its entire cross-section [7,20]. Visualizing the entire cross-section of the stomach can increase confidence in the quality of the observations.

Transducer Selection for Gastric Ultrasound

To obtain an optimal view of the gastric contents and antrum, first select abdominal imaging settings on the ultrasound machine. The selection of an ultrasound probe for gastric imaging is then chosen, guided by a combination of factors, including the patient's body habitus, the depth of the target anatomy, and the resolution required [21].

Both curvilinear and straight probes have been mentioned in the literature as effective for gastric imaging, supporting the versatility of ultrasound equipment in accommodating different clinical scenarios [21]. Our preference for the curvilinear transducer at our institution stems from its widespread applicability and consistent performance across various patient types. For the choice of probes, a low-frequency (2-5 MHz) curved array probe is generally adequate for most adult patients [1]. In pediatric cases, particularly for small children, or smaller adults, a higher frequency (5-13 MHz) linear probe might be more appropriate and can provide higher resolution [1]. While there is flexibility in the choice of probes for gastric ultrasound, it is crucial to select one that ensures adequate penetration for effective visualization of deeper abdominal structures like the aorta.

Obtaining Antrum Ultrasound Views

**Figure 1:**
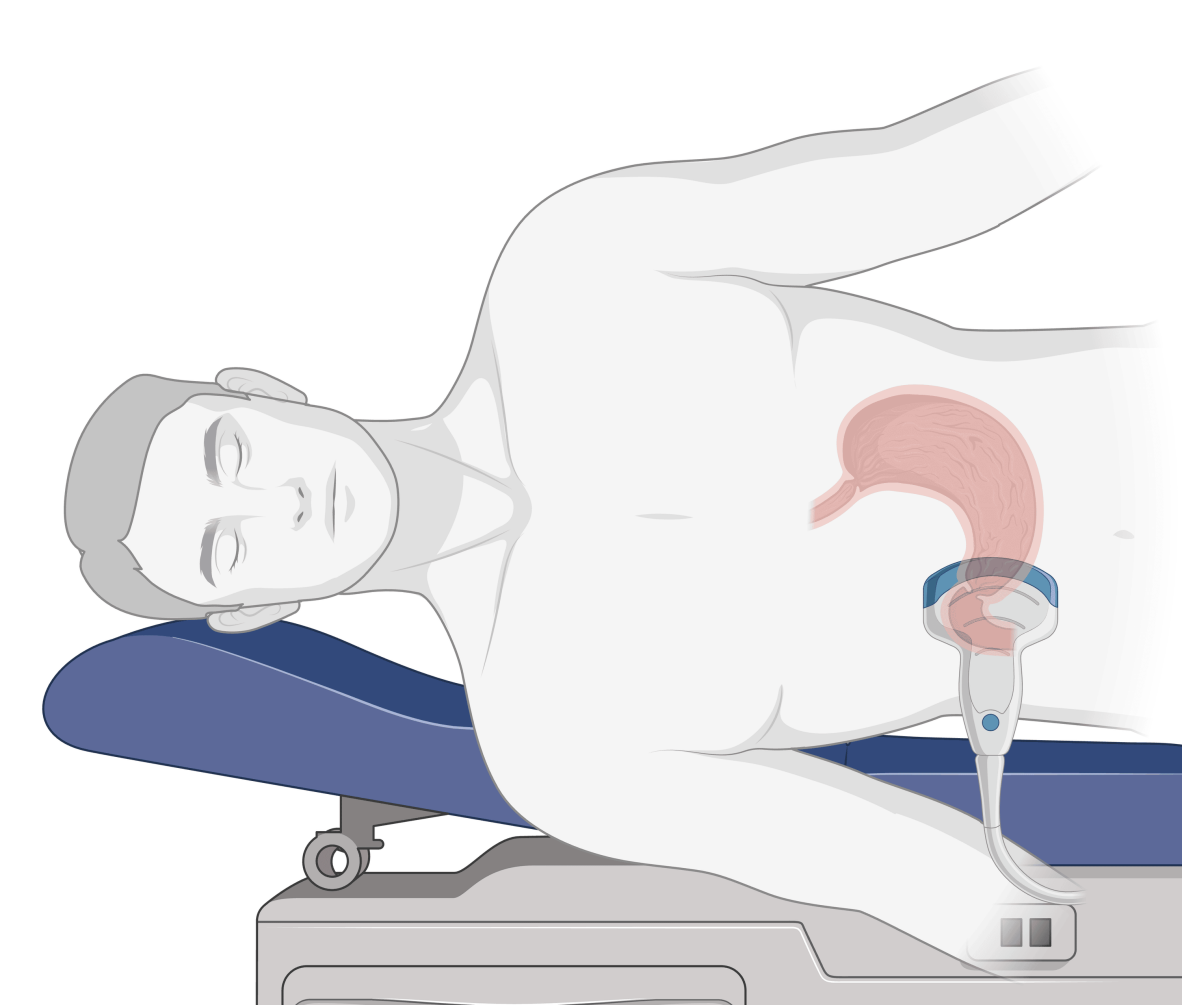
While the patient is in the right lateral decubitus position, the ultrasound probe is placed in the parasagittal plane just right of the midline for a parasagittal view of the gastric antrum Created with BioRender.com

**Figure 2:**
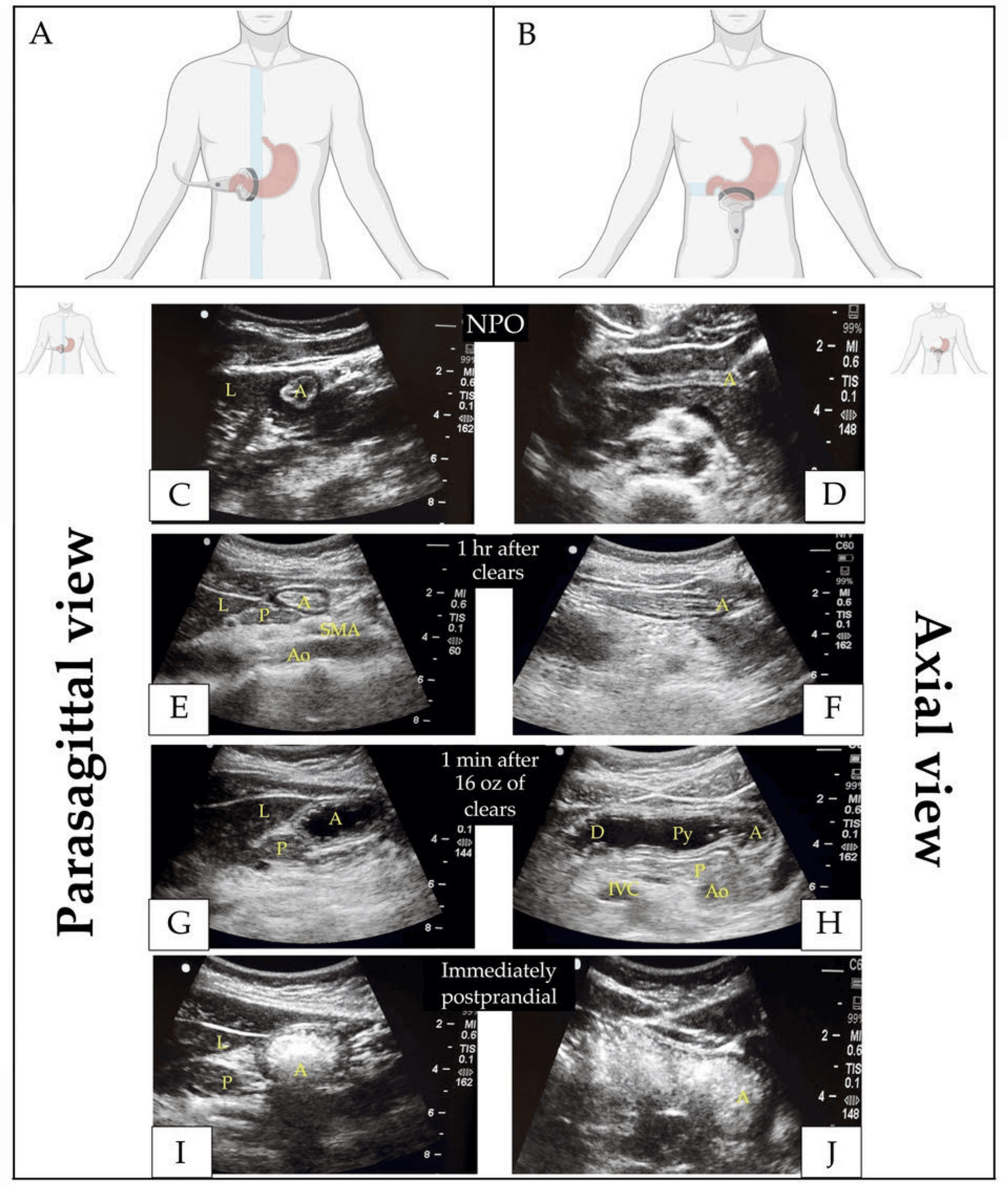
(A) Approximate ultrasound probe location and orientation for a parasagittal view of the gastric antrum; (B) Approximate ultrasound probe location and orientation for an axial view of the gastric antrum; parasagittal (C, E, G, I) and axial (D, F, H, J) views of the gastric antrum in: empty stomach state (C, D); 1 hour after ingestion of clear liquids (E, F); 1 minute after drinking approximately 500 mL (16 oz) of clear liquids showing characteristic expanded lumen with hypoechoic contents (G, H); and immediately following a meal with characteristic "ground-glass" appearance (I, J) Created with BioRender.com A: antrum; Ao: aorta; D: duodenum; hr: hour; IVC: inferior vena cava; L: liver; min: minute; NPO: nil per os; oz: ounce; P: pancreas; Py: pylorus; SMA: superior mesenteric artery

In preparation for imaging, the patient is placed first in the supine position and then in the right lateral decubitus position to allow gravity to direct any gastric contents to the antrum. With the appropriate application of ultrasound gel, the probe is typically placed in the parasagittal plane just right of the midline (Figures 1, 2). The transducer should be positioned on the patient so that the orientation marker points towards their head. The orientation marker on the ultrasound screen and cephalad structures should be adjusted so that they are both displayed on the left of the screen. The probe can also be placed in the axial plane, transversely across the abdomen (Figure 2). Table 1 and the discussion below compares the relative benefits of these two orientations. Once the probe is placed correctly and oriented as desired, the operator can adjust the depth, gain, and probe angle to obtain a clear image of the antrum.

**Table 1:** Comparison and overview of parasagittal and axial views in bedside gastric ultrasound* *[6,20,22]

	Parasagittal View	Axial View
Probe Orientation	Longitudinal, parallel to the sagittal plane of the body	Transverse, parallel to the transverse plane of the body
Probe Placement	Just right of the midline	Just right of the midline
Patient Position	Supine initially, then right lateral decubitus	Supine initially, then right lateral decubitus
Antrum Appearance	Empty antrum displays a "bullseye" pattern; stomach walls encircle contents when full	Looks like a "gloved finger"
Advantages	Clearly delineates the entire contour of the antrum. Best for estimating gastric volume and content overall	Used to locate the antropyloric-duodenal transition

Figure 2 shows stomach views in parasagittal and axial planes during four states: empty after adequate NPO (*nil per os*, fasting) time, one hour after clear liquids, one minute after 16 fluid ounces (approximately 475 mL) of clear liquids, and immediately postprandial after a meal to illustrate the characteristic findings in these situations. The appearance of any visible gastric lumen can give some indication of food type. Clear liquids will appear hypoechoic, possibly with a "starry night" appearance when there are air bubbles. Thicker liquids like yogurt or some soups may appear hyperechoic and homogenous, while solid food often presents a heterogeneous "frosted glass" appearance.

Only a roughly parasagittal probe orientation reliably provides the commonly recommended [6,7,20] full cross-section view. Larger volumes, indicated by an expanded lumen or any observable volume of solid food, indicate increased risk [22].

Parasagittal View

An appropriate parasagittal view is obtained when the antrum demonstrates a "bullseye" pattern (Figure 2 panels C, E, G, and I). The caudate and left lobes of the liver should be visible anteriorly and the head or neck of the pancreas posteriorly, and the aorta or inferior vena cava is often visible posterior to the pancreas (Figure 2) [19]. The antrum is best visualized in the parasagittal plane of the two views presented. This view should clearly delineate the entire contour of the antrum, making it particularly useful for evaluating the volume and nature of gastric contents, including quantitative estimation when that is desired [6,20,23]. The antrum is considered empty in this view if it appears as a small bullseye without an open lumen. The antrum is considered to contain food/drink if it appears to have an endocavitary lumen and distended walls [21].

Axial View

In the axial view, the empty antrum shows as a "gloved finger" without a fully defined cross-section (Table 1, Figure 2). We generally do not recommend sole reliance on the axial orientation as the contour of the antrum is often incomplete or vague in this view, especially when the stomach is full (Figure 2). This view might also be confusing when the gastric volume is low, as the anterior and posterior walls will be juxtaposed. The posterior wall may not be visible with a full stomach, especially one containing solid food. Although the axial view might not be as clear as the parasagittal view for gastric content evaluation, its different perspective can facilitate the location of the antropyloric-duodenal transition [6,20,23].

## Discussion

Current ASA Practice Guidelines for Preoperative Fasting are based on time since consumption of significant food types (i.e., solid food, milk/juice, breastmilk, clear liquids) and do not account for individual physiological or context-dependent variation in gastric emptying [10]. Integrating bedside gastric ultrasound into perioperative protocols enables verification of a patient's stomach's state and may ultimately stimulate revision of these guidelines. Time-based guidelines have been demonstrated to yield inconsistent results [22,23] due to multiple patient factors, including anxiety, medication administration, anatomical variation, prior surgery, and gastrointestinal infection [24]. Ultrasound-guided assessment allows for a more personalized approach relying on evidence directly from the patient, likely enhancing patient safety outcomes. The non-invasive nature of ultrasound, combined with its ability to provide real-time feedback, enhances patient comfort and understanding.

In an emergent situation, many practitioners assume that the stomach is full and proceed with a cuffed endotracheal tube, rapid-sequence intubation, and other techniques to guard against aspiration risk. A bedside determination that the stomach has minimal contents may permit other techniques that are preferred in some cases, such as a laryngeal mask airway and fiberoptic bronchoscope in the setting of reduced neck mobility or concern for cervical trauma.

While gastric content volume can be quantitatively estimated with ultrasound measurements and published formulae [19,20], attempts to arrive at a formal estimation or even obtain two views may save time to achieve an unnecessary (and perhaps optimistic) level of assumed precision in the estimate. As outlined below, a qualitative determination of the extent of gastric contents may be helpful.

We use a simple 3-point grading system proposed by Perlas et al. (Table 2) for routine clinical practice use [19]. Our concern for aspiration risk is highest for Grade 2 cases and somewhat reduced for Grade 1. We are reasonably confident that the stomach will likely be empty only with Grade 0 cases.

**Table 2:** Grading system for gastric content assessment using bedside ultrasound* *[21]

Grade	Description
0	Antrum appears empty in both supine and right lateral decubitus positions, indicative of an empty stomach.
1	Minimal fluid volume (<1.5 mL·kg^-1^) is detected only when the patient is in the right lateral decubitus position
2	Antrum visibly distended with fluid (≥1.5 mL·kg^-1^) in both supine and lateral positions, suggesting a full stomach

A common objection to this two-view examination arises in situations with time pressure. We have found that in emergent cases, an even faster categorical assessment based on a single parasagittal view of the antrum alone, especially in the right lateral decubitus position, can provide valuable information to help address whether stomach contents present significant aspiration risk [19]. Our recommendation for the right lateral decubitus position, when possible, in this situation arises from the fact that gastric contents will then typically pool in the antrum due to gravity.

As the popularity of medications such as Ozempic® (semaglutide), Trulicity® (dulaglutide), and other glucagon-like peptide-1 (GLP-1) receptor agonists grow for managing type 2 diabetes and promoting weight loss, the ASA has advised patients to pause these drugs one week before elective surgeries. Initial research shows that those who experienced gastrointestinal issues, such as nausea or vomiting with the concomitant use of GLP-1 agonists, had increased residual gastric contents. If a patient has not held their GLP-1 agonist medication, the ASA recommends the presumption of a "full stomach." Hence, the ASA suggests employing gastric ultrasound to evaluate stomach contents in these patients [25]. Should the ultrasound show an empty stomach, the procedure can be performed. However, if the stomach appears full or the ultrasound results are equivocal, surgery should be postponed or precautions taken for a full stomach, such as a rapid sequence induction.

Ultrasound's dependency on operator skill and experience can limit its applicability by slowing down the assessment in an emergent situation. This is highlighted by studies showing that a median of 24 cases was needed among anesthesiologists learning the procedure of gastric ultrasound to achieve a 90% success rate, with a learning curve that varied among individuals [26]. With adequate training and guidance, it is projected that anesthesiologists can achieve a 95% success rate in conducting bedside qualitative ultrasound assessments after approximately 33 examinations [26]. These findings emphasize the need for appropriate training or certification of practitioners involved in preoperative assessments. While the antrum is usually easily accessible and provides valuable information, a comprehensive understanding of gastric content and volume might require a more complete view of the stomach. Future research may explore integrating more advanced techniques, including 3D ultrasound imaging, to provide a more comprehensive assessment.

While gastric ultrasound represents a promising solution to the challenges of aspiration risk assessment, its integration into routine practice necessitates a multifaceted approach. This approach involves critical appraisal of existing guidelines, investment in practitioner training, and well-designed observational trials to evaluate this modality's accuracy and utility. As healthcare continues to evolve towards more personalized and evidence-based practices, technologies like ultrasound will play a pivotal role.

## Conclusions

This educational report highlights the critical role of bedside gastric ultrasound as an essential diagnostic tool in teaching and practicing anesthesiology, particularly in managing the risk of pulmonary aspiration. It can identify patients with increased gastric volumes and/or solid food material immediately before surgery. The parasagittal antral view, in the right lateral decubitus position when possible, is most valuable for assessing aspiration risk even without precise volume estimation. In cases where the nature of the emergency or patient condition limits movement, even a single view can be informative. With the proposed three-category grading system, practitioners can significantly enhance decision-making in elective and urgent surgical scenarios. All available evidence indicates that Grade 0 patients retain low or negligible volumes of gastric contents, and thus face a lower risk of gastric aspiration. Gastric ultrasound is invaluable for identifying patients with potentially high gastric volumes or solid food contents, thereby enabling more accurate stratification of aspiration risk.

The educational component, however, is crucial. We emphasize that there is a learning curve associated with gastric ultrasound mastery. This report serves as a guide, illustrating the necessity for comprehensive training to ensure effective utilization. Such training could improve patient safety and quality of care, particularly in emergent situations or unique patient populations where traditional fasting guidelines may not provide sufficient confidence. The integration of gastric ultrasound into clinical practice is poised to play a crucial role in personalized patient care. Healthcare professionals with this skill set will be more proficient at accurately assessing the risk of aspiration, thereby optimizing patient outcomes through more informed and tailored clinical decision-making.
