# Correction: Rapid Evaluation of Gastric Content With Ultrasound: An Educational Tool

**DOI:** 10.7759/cureus.c352

**Published:** 2025-10-09

**Authors:** Huynh Nguyen, Matthew R Paluska, Ricardo Falcon, Timothy R Petersen, Codruta Soneru

**Affiliations:** 1 Department of Medicine, The Keck School of Medicine of the University of Southern California, Los Angeles, USA; 2 Department of Anesthesiology, Rocky Vista University College of Osteopathic Medicine, Englewood, USA; 3 Department of Anesthesiology and Critical Care, University of New Mexico School of Medicine, Albuquerque, USA; 4 Office of Graduate Medical Education, University of New Mexico School of Medicine, Albuquerque, USA; 5 Department of Obstetrics & Gynecology, University of New Mexico School of Medicine, Albuquerque, USA

This article has been revised to correct Grade 0 in Table 2. Previously it read “Antrum appears empty in both supine and prone positions, indicative of an empty stomach.” This has been corrected to “Antrum appears empty in both supine and right lateral decubitus positions, indicative of an empty stomach.”

